# Genome-Wide Analysis of Family-1 UDP-Glycosyltransferases in Potato (*Solanum tuberosum* L.): Identification, Phylogenetic Analysis and Determination of Response to Osmotic Stress

**DOI:** 10.3390/genes14122144

**Published:** 2023-11-27

**Authors:** Yongchao Wu, Jie Liu, Baozhen Jiao, Tingting Wang, Sifan Sun, Binquan Huang

**Affiliations:** School of Agriculture, Yunnan University, Kunming 650504, China; wyc@mail.ynu.edu.cn (Y.W.); jieliu@mail.ynu.edu.cn (J.L.); jiaobaozhen@126.com (B.J.); jnwtt@sina.com (T.W.); sunsf236@163.com (S.S.)

**Keywords:** osmotic stress, potato, UDP-glycosyltransferase, expression pattern

## Abstract

Family-1 UDP-glycosyltransferases (UGTs) are the most common and functional glycosyltransferases in the plant world. UGT is closely related to plant growth and the response to abiotic stress. However, despite systematic research, our understanding of potato UGT genes is still unclear. In this study, we identified 174 potato UGT proteins based on their conserved plant secondary product glycosyltransferase (PSPG) motifs. Phylogenetic analyses were used to compare these proteins with *Arabidopsis* UGTs and other plant UGTs, and it was found that they could be clustered into 18 distinct groups. Patterns of intron gain/loss and intron phases within potato UGTs revealed highly conserved intron insertion events. The promoter *cis*-elements of these 174 *UGT* genes were systematically investigated. The promoter regions of these *UGT* genes are known to contain various classes of *cis*-acting compounds. These include elements that are light-responsive, phytohormone-responsive, and stress-responsive. Transcriptome data analysis established that 25, 10, 6, and 4 of these 174 *UGT* genes were specifically expressed in leaves, roots, stolons, and young tubers, respectively. The mannitol-treated transcriptomic data showed thirty-eight *UGT* genes were significantly upregulated. The quantitative real-time PCR results showed that the four genes were all responsive to osmotic stress under a 10% PEG6000 treatment. The results of our study provide a basis for clarifying the molecular mechanism of potato osmotic stress resistance and better understanding its function in the future.

## 1. Introduction

Glycosylation is one of the most important protein translational modification reactions and is the last step in the synthesis of natural compounds, which promotes the diversity and complexity of plant secondary metabolic reactions [1]. Glycosylation is catalyzed by glycosyltransferase enzymes (GTs) and catalyzes the transfer of sugar moieties from donor molecules to specific acceptor molecules (sugars, lipids, etc.) [2]. The latest data from CAZy show that GTs are classified into 114 families in different species by their 3D structure (fold GT-A, GT-B, or predicted GT-C) and their mechanism (reversal or retention) [3]. Family-1 GTs comprise the largest family out of all subfamilies [4].

Family-1 GTs are the most common and functional glycosyltransferases in the plant world [5], in which the donor chemical is uridine 5′-diphosphate. Analysis of the UGTs’ sequences shows that their extremely varied N-terminal region is able to detect a range of substrates [6]. There is a motif in the C-terminal region termed PSPG-Box which is responsible for combining UDP sugars [7]. The PSPG-box is a highly conserved region comprising 44 amino acids in the *UGT* of all studied plants [8]. As their amino acid sequence and conserved domain have been identified, UGTs are no longer mysterious in many species. In *Arabidopsis thaliana*, there are a total of 120 *UGT* genes that were divided into 14 groups by phylogenetic analysis (groups A to N) [9]. Ninety-six UGTs were identified in *Cicer arietinum*, and their intron–exon structure was analyzed [10]. In *Zea mays*, 147 UGTs were identified, and then a phylogenetic tree was constructed with 18 UGTs of *Arabidopsis* and 2 UGTs of rice. The UGTs were divided into 17 groups, including 14 conserved groups in *Arabidopsis* and 3 new groups [11]. Most recently, in *Gossypium hirsutum* [12], *Triticum aestivum* L. [6], and *Citrus grandis* [13], 274, 179, and 145 UGTs were identified, respectively.

UGTs are present in many species and play important roles in the growth of plants [14]. Studies have shown that UGTs recognize different substrates, such as, for example, flavonoids, terpenoids, auxin, salicylic acid, and sterols [15]. These molecules can be glycosylated to enhance their activity, solubility, chemical stability, or biological activity and promote storage and accumulation in plant cells [16]. Moreover, recent studies have shown that many glycosyltransferase genes are related to abiotic stresses. For example, in *Arabidopsis*, overexpression of *UGT75B1* genes increased the seed germination rate and seedling greening rate under salt and osmotic stresses [17]. Meanwhile, overexpression of *UGT79B2/B3* greatly increased plant resistance to drought, low temperatures, and salt stressors; conversely, double mutants of *UGT79B2* and *B3* were more vulnerable to unfavorable circumstances [18]. Zhao et al.’s research demonstrated that inhibiting *CsUGT78A14* reduced flavonoid accumulation and the reactive oxygen species scavenging ability, thus reducing the resistance of tea plants under cold stress. Inhibiting the expression of *UGT91Q2* reduces cold stress by reducing accumulation of nerolidol glucoside and the reactive oxygen species (ROS) scavenging capacity in *Camellia sinensis* transgenic plants [19]. The involvement of *AtUGT73C6* and *AtUGT78D1* in the production of flavonoid glycosides in *Arabidopsis* has been verified [20]. Summarizing the above, flavonoids are decisive in abiotic stress. On the other hand, plant UGTs catalyze ABA glycosylation. For instance, *UGT71B6* is able to identify (+)-ABA, the ABA enantiomer that occurs naturally [21]. Mutations in the *UGT71C5* gene enhance drought tolerance in *Arabidopsis* [22]. *UGT71B7* and *UGT71B8* are induced by ABA, NaCl, and mannitol [23]. These results show that *UGT* genes are involved in responding to stress by altering ABA glycosylation. 

Potato (*Solanum tuberosum* L.) is the world’s most important non-grain food crop and the fourth-largest crop in terms of production volume [24]. In previous studies, it was found that anthocyanin 5-O-glucosyltransferase (5-UGT) functions to modulate the process of potato tuber metabolism. The ectopic expression of *5*-*UGT* enhances the pathogen infection resistance of potato to *Erwinia* [25]. Meanwhile, its overexpression increases the level of glycosylated anthocyanidins in transgenic potato plants. However, a systematic investigation of *UGT* genes under abiotic stress in potato still needs to be carried out. In the process of potato cultivation, abiotic stresses, especially osmotic stresses, are not conducive to potato growth, for example, in a water-scarce or high-salinity environment, resulting in ROS accumulation, cell death, and plant yield inhibition [26]. Studies have demonstrated that flavonoids are essential secondary metabolites (SMs) that are crucial in preventing and reducing the harm caused by osmotic stress [27].

In this study, we identified 174 UGT proteins from potato that could be divided into 18 groups. Transcriptome data showed that 38 *UGT* genes were significantly induced by a mannitol treatment. Four *UGT* genes were selected for further qRT-PCR verification. These genes were upregulated by a 10% PEG6000 stress treatment, which indicates that these genes might participate in the osmotic stress response. Overall, our findings provide candidate *UGT* genes involved in osmotic stress for further functional study.

## 2. Materials and Methods

### 2.1. Identification of UGT Genes in Potato

The genome and genome annotation files (GFF3) of potato (estimated size: 785 MB with 56210 genes) were downloaded from Ensembl Plant (https://ensembl.gramene.org/Solanum_tuberosum/Info/Index, accessed on 17 October 2022). Using Tbtools (version No.2.007), a query was created using the 44 amino acid conserved sequences of the PSPG motif to search the potato genome database [28]. The expectation-value (E-value) cutoff was −5. The NCBI (https://www.ncbi.nlm.nih.gov/, accessed on 25 October 2022) was used to confirm each predicted potato UGT protein sequence.

### 2.2. Phylogenetic Analysis of Potato UGT Genes

Using MEGA-X’s ClustalW 10.0 software, multiple alignments of the potato UGT amino acid sequences were performed. With the bootstrap set to 1000 replicates and the neighbor-joining method enabled, the phylogenetic analysis was completed.

### 2.3. Chromosomal Locations

The chromosome position information of potato UGTs was obtained through the genome annotation files (GFF3). We also made use of Tbtools’ Drawing Atlas.

### 2.4. Intron Mapping

By identifying the intron splice locations, phases, and placements, the potato UGT intron map was created. Using CDSs and genomic sequences, the online Gene Structure Display Server 2.0 (http://gsds.gao-lab.org, accessed on 5 November 2022) was able to obtain the exon–intron structure and intron phases. After aligning every potato UGT, an amino acid sequence was created, and the introns were serially numbered in accordance with those places. The following were used to identify the intron phases: phase 0 corresponded to introns between two codons, phase 1 corresponded to introns after the first base in the codon, and phase 2 corresponded to introns after the second base in the codon [29].

### 2.5. Promoter Analysis of UGTs

The 1500 bp sequence before the CDS in UGTs, as the promoter region, was predicted using the PlantCARE website (http://bioinformatics.psb.ugent.be/webtools/plantcare/html/, accessed on 25 October 2023).

### 2.6. Plant Materials and Treatment

*S. tuberosum* L. Desiree (from the College of Agriculture, Yunnan University; it is a tetraploid) was used for analysis in this study. Four-week-old tissue culture plantlets’ stems were cut to a length of about 2 cm, and they were then placed in sterile glass jars filled with Murashige–Skoog (MS) liquid medium. They were placed in the culture room (light conditions for 16 h, 8 h of dark, and a temperature of 22 °C).

Four-week-old plants were cultivated in MS liquid medium containing 10% PEG6000 and were sampled at 0 h, 6 h, 12 h, and 24 h after treatment. 

### 2.7. Expression Analysis and Quantitative Real-Time PCR

RNA-seq data of the potato UGT genes were obtained from the published sequencing file [30]. The generated heat maps use 7 distinct tissues of RH (RH89039-16—material in transcriptome sequencing; it is a diploid), as well as the control and mannitol treatments with DM (DM1-3 516 R44—material in transcriptome sequencing; it is a diploid). 

For RNA extraction, the instructions provided in the reference kit (Takara, Dalian, China. code 9769) are used to isolate total RNA from a variety of potato tissues. Following the protocol, 0.5 μg RNA was used for reverse transcription reactions, and the Prime-Script RT reagent kit with a gDNA Eraser (Takara, Code No. 6110B) was used. Using a Bioer Technology FQD-96A and Tsingke 2×T5 Fast qPCR Mix (SYBR Green I), the qRT-PCR was performed, with three biological replicates. Elongation factor 1-α (Ef1α) served as the housekeeping gene in the qRT-PCR [31]. The 2^−ΔΔCt^ technique was utilized to ascertain the relative expression levels [32]. GraphPad prism 8.0 (https://www.graphpad.com/, accessed on 4 March 2023) was used to analyze the data and present them in graphs. Tukey’s pairwise comparison was used to test the significance. The relevant assay primers are included in Supplementary Table S7.

## 3. Results

### 3.1. Identification and Characterization of the UGTs 

Recently, the potato genome was sequenced to aid in the identification of potato gene families [30]. To identify the UGT family members in the potato, we used a conserved UGT domain of 44 amino acids called the PSPG-box to identify the UGT proteins in the potato genome. Blastp searches against the 56,210 potato protein models were conducted, and genes encoding identical proteins or sequences that were either too long or too short were eliminated [33]. A total of 174 UGT proteins were identified (Supplementary Table S1). Using the Ensemble potato sequence database and published sequencing files, detailed information was gathered about the potato *UGT* genes, such as the gene ID, transcript ID, length of the protein, molecular weight, isoelectric point, chromosome locations, splicing variants, and the putative function of the UGTs (Supplementary Table S1). The 174 *UGT* genes encoded proteins ranging in length from 352 (*PGSC0003DMG400017249*) to 506 (*PGSC0003DMG400011740*) amino acids, and the average length of the amino acids was 460. The gene molecular weight (MW) ranged from 39.428 to 57.037 kDa, and the isoelectric point (pI) ranged from 4.79 to 9.22. Their transcripts were encoded by 1-6 exons (*PGSC0003DMG400025532* contains 6 exons), and they had from 1 to 3 splicing variants. 

### 3.2. Phylogenetic Analysis of Potato UGT Genes

**Figure 1:**
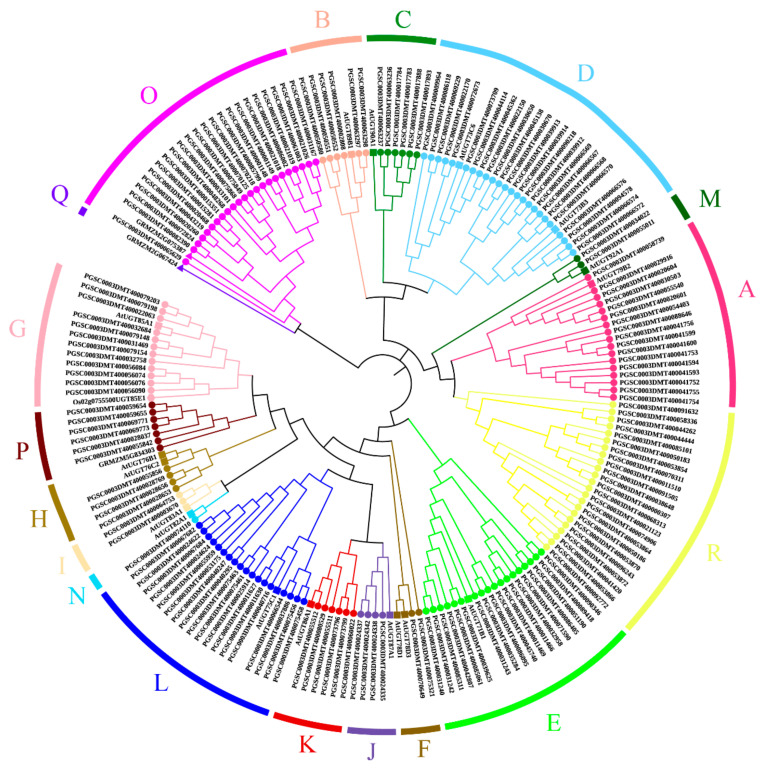
Phylogenetic analysis of the UGT proteins from potato, *Arabidopsis*, maize, and rice. A total of 174 UGTs were divided into 18 groups (groups A–R). Bootstrap values over 50% are indicated above the nodes.

To study the evolutionary relationships between potato, *Arabidopsis*, maize, and rice UGT proteins, a phylogenetic tree was constructed with the four species (174 UGTs of potato, 17 UGTs (AtUGT79B2, AtUGT89B1, AtUGT90A1, AtUGT73B3, AtUGT71B1, AtUGT78D3, AtUGT85A1, AtUGT76B1, AtUGT83A1, AtUGT87A1, AtUGT86A1, AtUGT75C1, AtUGT92A1, AtUGT82A1, AtUGT73C6, AtUGT78D1, AtUGT76C2) of *Arabidopsis*, 3 UGTs (GRMZM2G075387, GRMZM5G834303, GRMZM2G067424) of maize, and 1 UGT (Oso2g0755500UGT85E1) of rice (Supplementary Table S2)). Phylogenetic analysis was used to divide the 174 UGT proteins into 18 groups (Figure 1). The distribution of all UGT proteins in each branch of the phylogenetic tree was uneven. It was found that 23 and 6 UGTs were clustered into two new groups, named groups O and P (Figure 1). In addition, we named a novel separate group R (Figure 1). There was only the *S. tuberosum* UGT (StUGT) protein in group C, and it was more abundant. Some of these groups have only StUGTs and *Arabidopsis* UGTs, where the specific distribution is as follows (total, potato, *Arabidopsis*): group D (26, 24, 2), group E (19, 18, 1), group L (19, 18, 1), group A (18, 17, 1), group I (3, 2, 1), group N (2, 1, 1), and so on. Group G also had the rice UGT (total, potato, *Arabidopsis*, rice): Group G (14, 12, 1, 1). Groups O and P contain potato, *Arabidopsis*, and rice UGTs. Group Q was not found to have potato UGTs.

The distribution of plant UGTs in the phylogenetic groups is summarized in Table 1. In regard to potato, groups D, O, and R expanded more than the other groups. Group N has only one member. Group D is the largest group of potato UGTs as it has 24 members and is clustered with functionally characterized AtUGT73C6 and AtUGT73B3 (Figure 1). In group F and group H, there are also two *Arabidopsis* UGTs in each, but they only have two and four potato UGTs, respectively. This indicates that group D may play an important role in potato.

### 3.3. Chromosomal Locations

**Figure 2:**
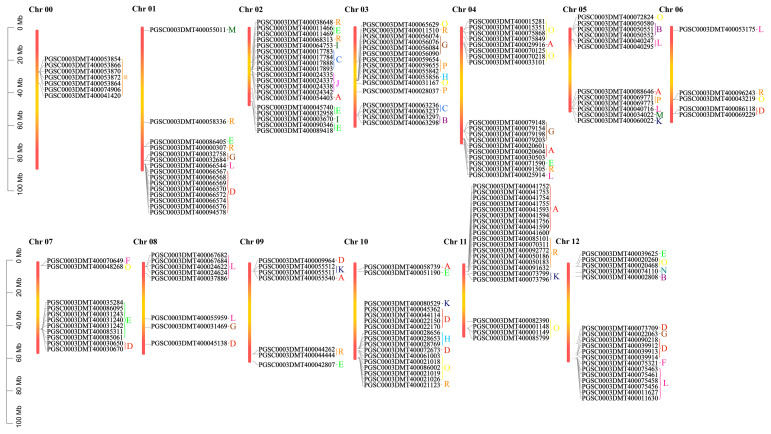
Chromosome distribution of potato *UGT* genes. Each chromosome is shown with its chromosome number at the top. Different colored letters represent different phylogenetic groups of potato *UGT* genes.

Based on the existing annotation information of the potato genome, the genetic map of the *UGT* genes on the potato chromosome was further studied (Figure 2). Potato has 167 *UGTs* distributed across all 12 chromosomes, with 7 *UGTs* located on chromosome 00 (Figure 2). This chromosome is different from other chromosomes because it contains only one group (group R). There were 21 *UGTs* on chromosome 11, which mainly contains group A, group R, group K, and group R. They are concentrated in the range of 0 mb to 20 mb, followed by 19 *UGTs* on chromosome 2. Chromosome 06 had the smallest number (5) of UGTs.

Combined with the above phylogenetic tree, it was found that there were different groupings on each chromosome (except for chromosome 11). Chromosome 12 in particular contains eight groups (groups B, D, E, F, G, L, N, and O) (Figure 2). Group R, consisting of 23 potato *UGTs*, had 2, 2, 1, 1, 1, 2, 1, and 6 members located on chromosome 01, 02, 03, 04, 06, 09, 10, and 11. Group D, consisting of 24 potato *UGTs*, had 8 members located on chromosomes 01 and 2, 2, 1, 1, 5, and 5 members on chromosomes 06, 07, 08, 09, 10, and 12 (Figure 2).

### 3.4. Analysis of Intron Gain/Loss Events

**Figure 3:**
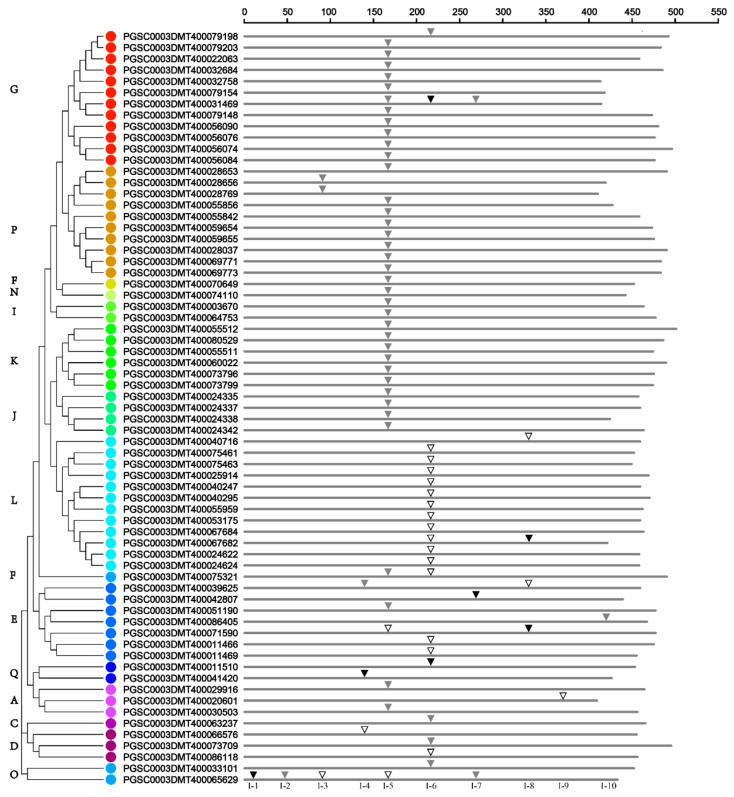
Distribution of introns among 67 *UGT* genes in potato. According to the alignment of their amino acid sequences encoded by the *UGT* genes, the introns are mapped and numbered. Intron phases 0, 1, and 2 are indicated by open inverted triangles (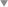), slash filled inverted triangles (▽), and black inverted triangles (▼), respectively. The phylogenetic relationships of potato UGT proteins can be seen on the left, and different phylogenetic groups are distinguished by colored dots.

To investigate the evolutionary relationships within the potato *UGT* gene family, the gene exon–intron structure was analyzed. Among the 174 sequences, 107 have no introns, while 61 and 4 have one and two introns each, respectively. In addition, *PGSC0003DMT400031469* contains three introns and *PGSC0003DMT400065629* has five introns (Figure 3). At least 10 intron insertion events occurred, numbered I-1 to I-10 according to their positions. Highly conserved introns were observed for intron I-5, which is contained in 39 (58%) potato *UGTs* belonging to groups A, E, F, G, I, J, K, N, and P (Figure 3). In addition, intron I-6 is concentrated in 12 sequences of a subgroup of group L. There were clearly intron gain/loss events that occurred in potato *UGTs*. Members from groups F, G, I, J, K, N, and P all gained intron 5, except *PGSC0003DMT400079198*, which lost intron 5 but gained intron 6. Meanwhile, for *PGSC0003DMT400031469*, it has intron 7 in addition to intron 5 and intron 6 (Figure 3).

Among the total 77 introns detected in potato *UGT* genes, 21, 49, and 7 were in phases 0, 1, and 2, respectively (Figure 3 and Supplementary Table S3). For the highly conserved I-5, only two were in phase 0, and phase 1 accounted for 94% of all introns. For introns I-6, 71% of *UGTs* were in phase 0. According to these results, the highly conserved introns were all in the same intron phase, which is in accordance with the view that most of the conserved introns are ancient elements, and their phases tend to be stable [36].

### 3.5. Expression Profiles of UGTs in Different Tissues

**Figure 4:**
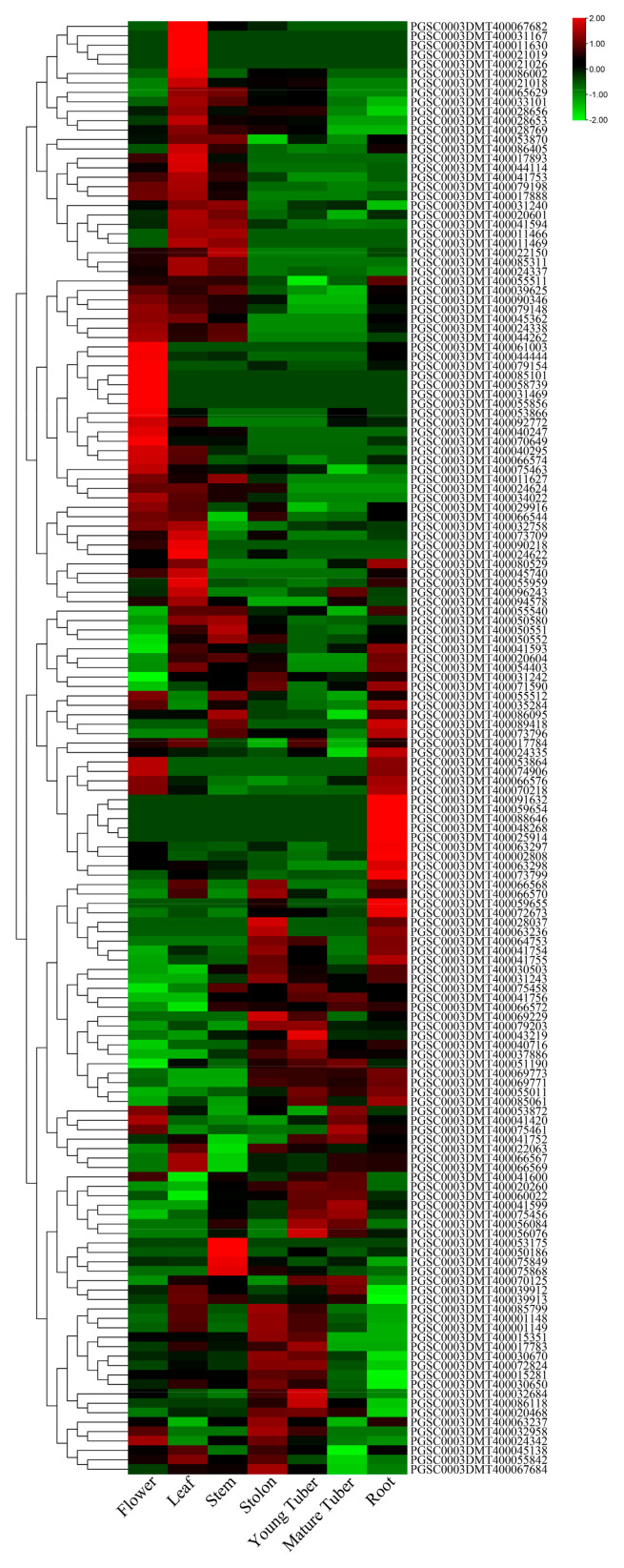
Distribution of *UGT* genes in potato tissues. Genes with zero expression in all seven tissues were removed.

To determine the expression patterns of potato *UGT* genes in different tissues, we used published RH transcriptome sequencing data [30]. The seven different potato tissues analyzed were from the flower, leaf, stem, stolon, young tuber, mature tuber, and root (Figure 4). Out of the 174 potato *UGT* genes, 154 *UGTs* were expressed in potato tissues (accounting for 88.5%) (Supplementary Table S4). Additionally, 25 *UGT* genes (accounting for 14.3%), 10 *UGT* genes (5.4%), 6 *UGT* genes (3.4%), and 4 *UGT* genes (2.2%) were specifically expressed in the leaves, roots, stolons, and young tubers (Figure 4 and Supplementary Table S4). For example, in all *UGTs*, *PGSC0003DMG400017508* was the most expressed in the roots but was not expressed in young tubers. *PGSC0003DMG400011740* and *PGSC0003DMG400000432* were expressed at high levels in the stolon and at low levels in other tissues. These results indicate that the special expressed genes might play an important role in leaves, roots, stolons, and young tubers, respectively.

### 3.6. Promoter Cis-Elemental Analysis

A promoter sequence determines the spatiotemporal expression pattern and level of genes. A promoter analysis of genes can help to explore their potential functions. Therefore, a 1500 bp sequence prior to the CDS from the StUGT genes was used as a promoter region for cis-element analysis. We found that the promoters of 174 UGT genes contain many kinds of cis-responsive elements, including light-responsive elements (chs-CMA1a, GT1-motif, Box 4, G-box, AE-box, TCT-motif, AAGAA-motif, etc.), phytohormone-responsive elements (TCA, TATC-box, ABRE, CGTCA-motif, TGACG-motif, etc.), and stress-responsive elements (LTR, MBS, ARE TC-rich repeats, etc.). A total of 68 UGT genes contained 7–10 light-responsive elements, and 105 UGT genes contained 1–5 light-responsive elements. PGSC0003DMG400007981 did not contain any light-responsive elements. In total, 159 UGT genes contained 1–5 plant hormone-responsive elements, and 8 UGT genes contained 6–7 elements (7 genes did not contain these elements). And 140 UGT genes contained 1–4 stress-responsive elements, and 34 UGT genes did not contain these elements. The above results suggest that these UGT genes might be involved in the abiotic stress response (Supplementary Table S5).

### 3.7. Expression Analysis under Abiotic Stresses

**Figure 5:**
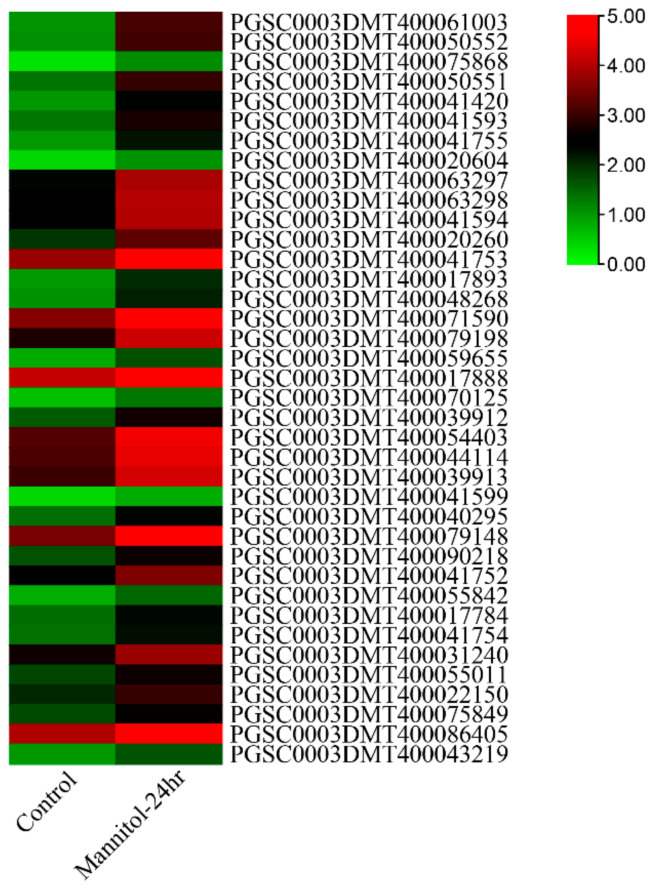
Expression profiles of potato *UGT* genes after 24 h mannitol treatment.

**Figure 6:**
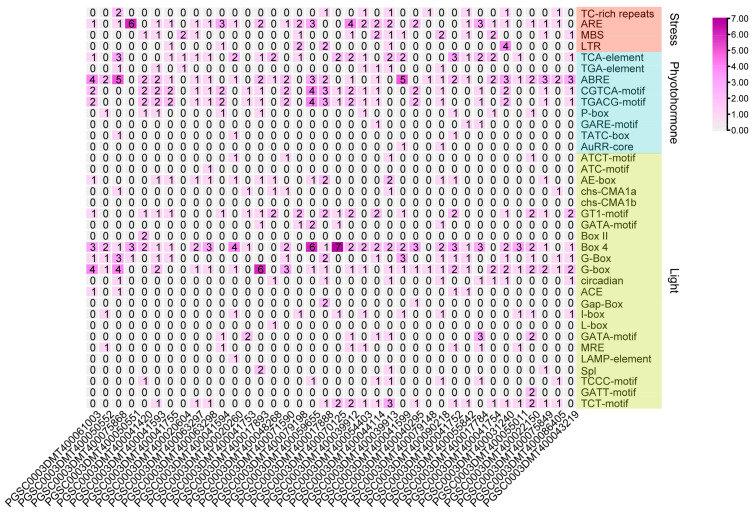
*Cis*-element analysis of *UGTs* in potato—under mannitol treatment for 24 h, the expression level was upregulated (absolute log2 fold change > 1). *Cis*-elements are mainly divided into light, pressure, and hormone-responsive elements. The number represents the color intensity.

**Figure 7:**
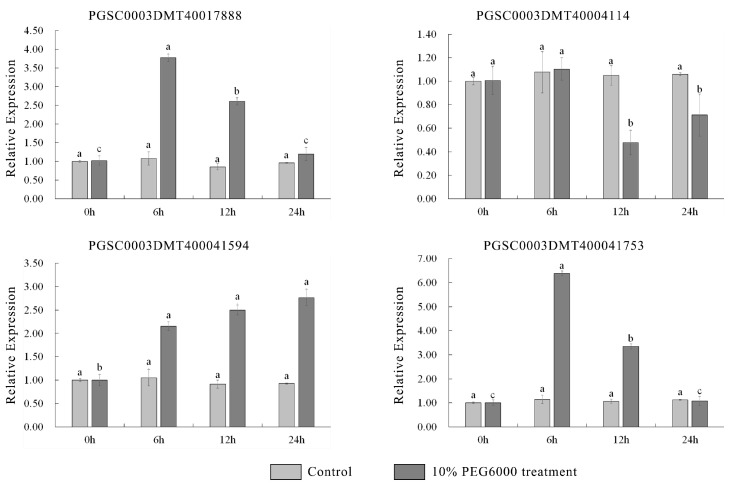
qRT-PCR results under 10% PEG6000 treatment. The error bars indicate the standard errors of three biological replicates. Significant statistical differences (*p* < 0.05; Tukey’s) across different time points are denoted by the lowercase letter(s) above the vertical bars.

Water loss from plant cells under drought conditions leads to osmotic stress and oxidative stress. To identify osmotic stress-related *UGT* genes, we analyzed the expression pattern of *UGTs* by using the public transcriptome under 260 μM mannitol stress. A total of 38 of the 174 *UGT* genes were significantly upregulated (absolute log2 fold change > 1) (Figure 5 and Supplementary Table S6). The promoter *cis*-elements of these genes also had an abundant number of photo-responsive elements. A total of 32 *UGT* genes contained 4-10 light-responsive elements, 35 *UGT* genes contained 2-5 hormone-responsive elements, and 32 *UGT* genes contained 1-4 stress-responsive elements, while 8 *UGT* genes did not contain these elements (Figure 6 and Supplementary Table S5). The top two genes with the highest expression level and the two genes with anthocyanidine rhamnosy1-transferase function were selected for RT-qPCR verification. These genes included: *PGSC0003DMT400041594*, *PGSC0003DMT400041753*, *PGSC0003DMT400017888*, and *PGSC0003DMT400044114*. As shown in Figure 7, the expression of *PGSC0003DMT400041594*, *PGSC0003DMT400041753*, and *PGSC0003DMT400017888* was significantly induced by 10% PEG6000, and peaked at 24 h, 6 h, and 6 h, respectively. Unexpectedly, the expression pattern of *PGSC0003DMT400044114* was the reverse of what was anticipated. These results indicate that the four *UGT* genes selected might participate in the osmotic stress response. 

## 4. Discussion

Due to their important roles in SM biosynthesis, the regulation of cell homeostasis, and the detoxification of xenobiotics, UGTs in plants have been a source of great interest [37]. Studies have demonstrated that in *A. thaliana* and *O. sativa*, the UGT family accounts for the majority of *GT* genes (>25 and >35%, respectively) [9]. In addition, UGT has been identified in many other plants, including *C. grandis* [21], *L. usitatissimum* [29], *Z. mays* [11], *V. vinifera*, *Malus domestica*, *Populus trichocarpa*, *Glycine max*, and *Mimulus guttatus* [34]. Phylogenetic analysis shows that similar sequences probably have the same ancestor, share the same structure, and have a similar biological function. However, no clear analysis of *StUGTs* was previously conducted in potato.

This study identified 174 *UGT* genes in potato, accounting for approximately 0.3% of potato proteins. According to the phylogenetic analysis, the potato UGTs can be grouped into 18 groups, including 14 highly conserved groups (A to N) and 4 newly discovered groups (O, P, Q, and R) (Figure 1). By clustering the rice UGT of group Q with the dicotyledonous potato UGTs, it was found that there was no clustering in group Q. However, tomato UGTs were also not classified as group Q [35]. This is consistent with previous research that found that group Q may exist only in monocotyledons [13]. Compared to other plants, potato and tomato have more extended group O members. This suggests that group O members may have significant functions within the Solanaceae family. A novel group containing 23 UGTs was also identified in our analysis, and we named it group R. According to the previous studies, the number of UGT groups varied significantly among various plant species, and in most plant species, group E has the largest number of UGT members. However, in the potato genome, group E ranks third in the number of members. Group D, which now comprises 24 genes (13.7%) of the putative UGT genes, has grown to be the largest group. In group D, StUGTs were mostly clustered on chromosomes 1, 10, and 12. In addition, Several UGT73s (belonging to group D) were functional in catalyzing the glycosylation of (iso)flavonoids and in the biosynthesis of anthocyanins [37,38]. Previous studies demonstrated that flavonoid overaccumulation was essential to enhance tolerance to drought stress [39]. In tomatoes, group D is mainly involved in tomato detoxification [35]. Therefore, group D may be involved in the abiotic stress resistance of plants. This is consistent with priest DM’s research, where UGTs were found to be involved in the biosynthesis of plant natural products such as flavonoids, phenylpropanoids, terpenes, and steroids, as well as in the regulation of plant hormones [21].

The occurrence of intron gain and loss events, as well as the phases and positions of introns in relation to protein sequences, provide key evolutionary clues [40]. Intron mapping of 174 peach *UGTs* revealed that 61% lacked introns, which is higher than the number of *UGT* genes lacking introns in maize (60%) [11] and *Arabidopsis* (58%) [41]. In potato *UGT* genes, ten intron positions have been identified, with I-5 being the most prevalent intron (Figure 3). Intron 5 was observed in most members of groups A, E, F, G, I, J, K, N, and P. In peach, maize, and black cottonwood [8,11,38], intron 5 is considered the oldest intron. The second most highly conserved intron was observed for I-6, which was mainly concentrated in group L of potato. A large number of introns in I-5 were in phase 1, while many introns in I-6 were in phase 0, and phase-0 and -1 introns outnumbered phase-2 introns. It appears that most conserved introns keep their phases stable [36].

Expression analysis was carried out with published RH/DM transcriptome sequencing data. It was found that 25, 10, 6, and 4 of these 174 UGTs were specifically expressed in leaves, roots, stolons, and young tubers, respectively. In other species, *1G091000* and *1G091100* are specifically expressed in peach blossoms and have been identified as playing important roles in the biosynthesis of anthocyanin [42]. These findings imply that genes expressed particularly in potatoes may be crucial to the growth and development of potatoes. In total, 62% were expressed in the stolon, 55% were expressed in the young tuber, and 48% were expressed in the mature tuber (Figure 4 and Supplementary Table S4). These observations suggest that there is a decline in secondary product metabolism during the maturation of potato tubers. 

*UGTs* respond to abiotic stresses such as mannitol and PEG, thereby reducing damage caused by abiotic stresses. Under mannitol treatment, 38 UGTs were upregulated more than twofold (Supplementary Table S6). Analysis of the elements on the promoter of these genes showed that there were abundant light-, stress- and hormone-responsive elements. The presence of these elements further provides the basis for these genes to respond to stress. For example, the down-expression of *UGT71C5* increased drought tolerance in transgenic plants, suggesting that *UGT71C5* may play a major role in coping with hormonal stress [22]. Previous research has shown that ROS accumulation is one of the earliest cellular responses induced by osmotic stress [43], and anthocyanins are a type of important SM that helps eliminate and reduce the active oxygen damage caused by osmotic stress directly or indirectly [40]. It has been shown that all anthocyanins identified in *Arabidopsis* contain at least one glycan group, and glycosylation catalyzed by UGTs is the final step in anthocyanin biosynthesis [44,45]. Therefore, four genes were selected for qPCR validation. The results show that the expression levels of *PGSC0003DMT400017888*, *PGSC0003DMT400041594*, and *PGSC0003DMT400041753* were significantly upregulated under the 10% PEG6000 treatment. Unexpectedly, the expression of *PGSC0003DMT400044114* was significantly downregulated. Meanwhile, in the phylogenetic analysis, PGSC0003DMT400041594 and PGSC0003DMT400041753 were clustered with AtUGT79B2, which contribute to cold, salt, and drought stress tolerance via modulating anthocyanin accumulation [18]. PGSC0003DMT400044114 was clustered with AtUGT73B3, which participates in the regulation of redox status and general detoxification of ROS-reactive SMs [46]. Thus, we speculate that *PGSC0003DMT400041594* and *PGSC0003DMT400041753* may protect plants from osmotic stresses through glycosylation of flavonoids (e.g., anthocyanin), and thus the accumulation of products that scavenge excess ROS in the plant, with PGSC0003DMT400044114 being involved in the negative regulation of the redox state and general detoxification of ROS-reactive SMs.

## 5. Conclusions

In summary, we provided the first thorough account of the *UGT* gene family in potato. The phylogenetic analysis revealed that 18 subfamilies might be formed from the 174 StUGT proteins (groups A to R). We further analyzed through intron mapping the promoter *cis*-elements and the expression pattern of the UGTs in potato under osmotic treatment. Selected genes were able to respond to osmotic stress. The functions of *PGSC0003DMT400017888*, *PGSC0003DMT400044114*, *PGSC0003DMT400041594*, and *PGSC0003DMT400041753* can be further investigated by genetic engineering under osmotic stress. The findings of this study contribute to furthering functional research and give insight into the history and possible uses of *StUGT* genes.

## Figures and Tables

**Table 1:** The number of UGTs in different phylogenetic groups.

UGT Group	*A. thaliana* ^a^	*Prunus* *persica* ^d^	*Malus × domestica* ^a^	*Vitis* *vinifera* ^a^	*Linum* *usitatissimum* ^b^	*Oryza* *sativa* ^a^	*Z. mays* ^c^	*Solanum* *lycopersicum* ^e^	*S. tuberosum* L.
A	14	10	33	23	16	14	8	26	17
B	3	2	4	3	5	9	3	2	6
C	3	4	7	4	6	8	5	2	6
D	13	19	13	8	21	26	18	18	24
E	22	29	55	46	22	38	34	18	18
F	3	4	6	5	1	–	2	2	2
G	6	34	40	15	19	20	12	11	12
H	19	9	14	7	6	7	9	5	4
I	1	5	11	14	9	9	9	2	2
J	2	7	12	4	4	3	3	1	4
K	2	7	6	2	5	1	1	5	6
L	17	18	16	31	19	23	23	18	18
M	1	14	13	5	3	5	3	3	2
N	1	1	1	1	1	2	4	1	1
O	–	1	5	2	–	6	5	25	23
P	–	4	5	11	–	9	1	4	6
Q	–	–	–	–	–	–	7	–	–
R	–	–	–	–	–	–	–	–	23
Total	107	168	241	181	137	180	147	143	174

^a^ Data from Caputi et al. [34]. ^b^ Data from Barvkar et al. [29]. ^c^ Data from Li et al. [18]. ^d^ Data from Wu et al. [13]. ^e^ Data from Yu et al. [35].

## Data Availability

Data are contained within the article and Supplementary Materials.

## Supplementary Materials

The following supporting information can be downloaded at: https://www.mdpi.com/article/10.3390/genes14122144/s1; Table S1: Characteristics of Family-1 UGTs from *S. tuberosum* L.; Table S2: *Arabidopsis* and other *UGT* genes used for the identification of the phylogenetic groups; Table S3: Intron information of 67 potato *UGT* genes; Table S4: FPKM values in RH tissues; Table S5: Analysis of cis-elements of *UGT* gene promoters in potato; Table S6: Upregulated *UGTs* under mannitol treatment; Table S7: Information about primers used for qRT-PCR.
